# Looking to the future: should ‘prognosis’ be heard as often as ‘diagnosis’ in medical education?

**DOI:** 10.1080/14739879.2015.1101863

**Published:** 2015-12-31

**Authors:** Peter Croft, Geert-Jan Dinant, Peter Coventry, Kevin Barraclough

**Affiliations:** ^a^ Arthritis Research UK Primary Care Centre, Keele University, Keele, UK; ^b^ Department of Family Medicine, Maastricht University, Maastricht, The Netherlands; ^c^ School of Medicine, Keele University, Keele, UK; ^d^ School of Social and Community Medicine, University of Bristol, Bristol, UK

Much medical education is concerned with principles of disease diagnosis and treatment. Much medical research is, as a result, concerned to inform those principles with new understanding about the causes and mechanisms of disease and how best to reach a diagnosis and prescribe effective treatments linked to the diagnosis.[1] Primary care education seeks to develop expertise in understanding the patient as a person at the centre of this clinical activity, and in the decision-making, communication and care that must frame a diagnosis and effective treatment for an individual patient.

Skills in history taking and examination, knowledge of differential diagnosis, and the use of investigations are taught as means to overcome diagnostic uncertainty and guide decisions about care for patients such as the person with new onset headache who may have a subarachnoid haemorrhage. Primary care education also aims to help the clinician learn how to handle uncertainty when further pursuit of a disease diagnosis is unlikely to influence the choice of treatment or alter the patient’s outcome. Concluding that a patient has simple or mechanical low back pain may not supply a distinct and definite pathological diagnostic label, as compared with, say, diagnosing lung cancer or polymyalgia rheumatica, but it does indicate a low probability of avoidable serious outcomes and provides an alternative to ‘disease diagnostic’ labels.[2] By contrast, a male aged 63 years with new onset loose stools and rectal bleeding may benefit from the search for a diagnosis because it leads to treatment which improves his likelihood of surviving with good health in the future. Diagnosis is of questionable relevance when it arbitrarily labels the patient as having a disease, and is not intimately linked with prognosis – such as a diagnosis of mild hypertension in low cardiovascular risk individuals [3] – or if it causes harm because it leads to excessive or inappropriate treatments and unnecessary investigations, such as a diagnosis of bone fragility in a patient with low hip fracture risk,[4] the phenomenon of overdiagnosis.[5]

A broader framework beyond disease diagnosis for teaching and thinking about such decision-making is provided by the science and art of prognosis,[6] which asks whether a decision will affect an individual patient’s future outcome. Disease diagnosis is important when it resolves uncertainty and improves the patient’s prognosis, most obviously in acute illnesses such as the significant unexplained breathlessness in a 40-year-old patient which could signify pulmonary embolism. For many patients, however, diagnosis alone does not provide sufficient knowledge and evidence about likely future health and quality-of-life status to guide care.[7] The diagnosis of coronary heart disease, for example, has been subsumed by different classifications defined by ECG patterns and blood markers, which carry very different prognoses and implications for treatment.[8] A patient in her 80s with a range of chronic medical conditions may have a likelihood of future dependency, hospitalisation and death most strongly predicted by inadequate diet, excessive medication and poor status of her feet, all of which can be altered to improve her prognosis.[9]

The increasing volume of information and research about a person’s risk of future health states, from starting points such as blood pressure or blood sugar or genetic markers, or psychological and social factors, adds fuel to the idea that prognosis should take a prominent place beside diagnosis as the basis for decision-making in clinical practice, drawing on the patient’s own wishes and values for the future, in order to deliver personalised useful interventions targeted to improve the patient’s likelihood of achieving those desired future outcomes.[8]

The perception that prognosis and prognosis research have growing roles to play in clinical practice implies that medical education at both undergraduate and postgraduate level needs to incorporate teaching about prognosis – how to estimate it, and express, think and communicate about it. This is not only about statistical evidence and its interpretation (‘the absolute risk of future stroke in this patient with atrial fibrillation is …. but the risk of serious side effects of treatment to reduce this risk and improve prognosis is ….’), but also about how to incorporate and apply such evidence in practice, alongside learning the classic lessons of diagnosis and treatment. This carries a number of challenges for primary care education, which are considered below, but first some reflections on the concepts of diagnosis and prognosis in primary care.

## The concepts

Diagnosis is a method of classification.[1] The child with a fever can be classified according to the different pathological causes of the fever, and treatment linked to the cause. The usefulness of the method is clear in situations where correct classification and treatment (of the feverish child with meningitis e.g.) averts serious outcomes. In primary care, less precise classifications are often useful in managing common conditions which do not have serious outcomes, such as the child with a self-limiting upper respiratory tract infection, for whom further investigations and antibiotics do not alter the outcome.

Prognosis offers another method of classification. Patients can be classified by the likelihood of what is going to happen in the future. ‘Your pain is going to get better’. ‘This infection will run its course’. This patient has mild prostatic symptoms, low PSA levels and is otherwise well – discussion about management can proceed on the basis that the risk of serious outcomes in the future is low.

The two methods are intimately related in practice. The judgement on whether a diagnostic label is important and useful or not (an X-ray report of cervical spondylosis in an older patient with neck pain e.g.) relates to whether patients with the diagnosis have different outcomes compared with patients who have neck pain and no cervical spondylosis on X-ray, and whether diagnosis-plus-treatment has any additional benefits or risks compared with no treatment. The availability of new CT imaging to detect pulmonary emboli offers important opportunities for diagnosis in patients with high clinical suspicion of emboli, but its widespread use also reveals minor abnormalities, treatment of which may not benefit and may result in harm to the patient.[10]

In primary care, time often helps resolve uncertainty through the processes of diagnosis, prognosis and treatment. As time passes (patient taking time to present, GP waiting to see what transpires), diagnosis and prognosis may both become clearer. The usefulness of a diagnostic label at any one time is whether action taken on the basis of the label (hypertension, depression) improves patient prognosis. The usefulness of the prognosis label is in identifying those patients (high risk of a poor outcome) where intervention is helpful, and those patients (low risk of a poor outcome) where overtreatment, ineffective treatment or dangerous treatment can be avoided.

## Education challenge 1: Introducing prognostic information to help clinical decision-making following a diagnosis

The first challenge for clinical education is how best to avoid undermining core tenets of practical clinical training with too much talk of uncertainty or by introducing prognosis as ‘anti-diagnosis’. In textbooks of clinical practice,[1] the classical triad is diagnosis-prognosis-treatment. Education and clinical training for primary care, in particular, needs to demonstrate the link between diagnosis and prognosis (diagnosis is important when it affects prognosis, and prognostic information and judgement can replace pathological diagnosis if the latter does not affect patient outcome), whilst instilling knowledge and facilitating the development of experience and expertise for the clinician to make useful diagnoses. Using examples focused on individual patients and their presenting problems (see atrial fibrillation below e.g.) links pragmatic clinical skills with evidence that each step in the decision-making process (diagnosis, prognosis and treatment) should lead either to improvement or at least to non-worsening of the patient’s future outcome.

Atrial fibrillation is a physical sign that can present in the context of presenting symptoms, or as a chance finding, or as the result of systematic screening. Diagnostic questions seek possible pathological reasons for this finding which vary from patient to patient. If there are no underlying conditions which need to be managed and treated, decision-making focuses on treatment of atrial fibrillation as a risk factor for future conditions (e.g. stroke) linked to disability and death. Shared decisions with patients can draw on information and guidance such as those of the UK National Institute of Health and Care Excellence.[11] One section states

Do not offer stroke prevention therapy to people aged under 65 years with atrial fibrillation and no risk factors other than their sex (that is, very low risk of stroke equating to a CHA_2_DS_2_-VASc score of 0 for men or 1 for women).

This model of clinical practice is all about prognosis – characteristics of the person other than their atrial fibrillation are used to estimate the likelihood of a future outcome (stroke) and to help inform a decision about treatment (anticoagulation to lower the risk of future stroke).

## Education challenge 2: Introducing prognosis as a way to interpret and manage common symptoms

The classic clinical teaching is that symptoms are signifiers of an underlying pathological disease process that must be identified. The reality of primary care is that many symptoms are treated in their own right. Judgement about individual patient prognosis (what is likely to happen to this patient in the future?), and the necessary evidence to inform that judgement, guides decisions about whether to investigate or not and whether to pursue a pathological diagnosis or not. Putting together evidence and judgements about what might happen in the future under the label of ‘prognosis’ provides a way to think about care for patients in whom the pursuit of diagnosis for its own sake may not be helpful.

General medical textbooks have for some years accepted ‘low back pain’ as a chapter title in contrast to other chapters named by disease topic, because diagnostic studies show most back pain is not caused by serious disease and can be managed as a symptom. There is of course an important initial diagnostic story. Certain features of back pain (fever, neurological symptoms e.g.) may guide immediate decisions to investigate for rare but treatable life-threatening disease (e.g. infection) or to avoid serious outcomes (e.g. paralysis from a spinal tumour), and there is an evidence base to be explored about the usefulness of these features in identifying the patients with serious disease.[12] In the vast majority of low back pain patients who have no indication of serious underlying disease, there is a prognostic story. Patients with simple low back pain can be classified by characteristics indicating the probability that they will subsequently improve or not. Evidence suggests that decisions guided by this classification result in better patient outcomes (such as time to return to work) and less unnecessary health care.[13] By contrast, patients with simple back pain randomised to have MRI scans were shown to receive more diagnoses and interventions than patients who did not have scans, but patient outcomes of pain and disability at 1 year were the same in the two groups.[14]

What if symptoms persist? The clinical care of patients with persistent symptoms must incorporate the importance of not missing diagnoses that are treatable. Prognostic evidence is one source of information to help decision-making about further investigation by asking about likely outcomes in patients with particular persistent symptoms and characteristics in primary care (e.g. chronic fatigue), and considering indications for investigation and seeking a diagnosis as well as the risks and benefits of doing so. Prognostic information also supplies the basis for positive approaches in patients whose persistent symptoms have no clear pathological diagnosis, by informing distinctive forms of treatment depending on the risk of future outcome in such patients. Evidence about which patients with chronic fatigue will improve and who will respond to treatments such as supervised exercise [15] offers an alternative approach to the pursuit of diagnosis alone.

## Education challenge 3: Introducing prognosis as a component of primary care’s focus on the patient and their concerns

Undergraduates or postgraduates learning and reflecting about medicine in general, and about primary care in particular, gain extensive appreciation of the breadth of issues involved in patient care beyond diagnosis and treatment. Lifestyle, social, familial, cultural, psychological and ethical components of illness are not excluded by the diagnosis-treatment model. They form important parts of medical training.

However, these factors are also important and significant predictors of the outcome of care that are often, especially for patients with chronic conditions, more important and relevant than the pathological diagnosis and its treatment alone. Such broader information now also includes genetic, biomarker and physiological measurements. A prognostic framework that considers all predictors of patient-valued outcomes offers additional insight to targets for care, especially in older patients with multimorbidity. The clinical focus can extend to ‘what is the likely outcome in this patient given the whole range of information about this individual beyond the specific diagnosis and treatment?’ For example, medical and psychiatric comorbidities predict mortality in patients with diabetes,[16] and psychological status and pain in sites other than the knee predict outcomes in patients following total knee arthroplasty.[17]

Patients with new or persistent symptoms are likely however to seek decisions framed around a diagnosis – have I got it or not? If the evidence base and the doctor’s clinical experience place the patient in a group with a risk of a poor outcome unless diagnosis and early treatment are pursued (thunderclap headache, unexplained breathlessness), then diagnosis and prognosis go hand-in-hand. For many other patients who present in primary care, shared decisions with the patient not to pursue further investigations or diagnostic approaches can draw on prognostic evidence and intuition. Stratifying patients by prognostic risk can inform decisions to avoid further diagnostic tests which will make no difference to patients at low risk of poor outcome (e.g. exercise tolerance tests in low risk chest pain patients [18]) and identify patients to whom the results of efficacy trials (often carried out in high-risk patients) do and do not apply.[19]

The statement that ‘there is nothing seriously wrong’ may be couched and discussed in diagnostic-style language but is all about prognosis; it means that ‘The evidence indicates that there is no diagnosis to make that will affect your future good health and well-being’ or ‘An Xray at this stage is very unlikely to reveal anything that will affect treatment or your future health’. Time may be important here. Agreement with a patient to return if a symptom persists is an acknowledgement that persistence may identify the patients as belonging to a group with a higher risk of a poor outcome. Crucial also is the question of what the patients themselves want or value.

These are not new learning concepts. Primary care education and research have long emphasised the need for practical alternatives to a model of clinical practice that only judges success by the revelation of the pathological cause of a patient’s symptoms. The implication of a prognostic framework for education about practice, however, is that all decisions about diagnosis, screening, investigation, referral or treatment should consider whether the decision will lead to changes in the individual patient’s likely outcome and whether this will be useful to the patient.

## Education challenge 4: Introducing uncertainty and the science of risk

The discussion and examples above illustrate where the concept of prognosis can build on or enhance the ‘diagnosis-treatment’ model without usurping or upsetting it. However, there are wider implications for medical education and training if more space and attention is to be given to a prognostic model of practice. One purpose of medical education is to provide a basis for practical decision-making in situations of uncertainty, and the science of diagnosis and evidence-based treatments contributes importantly to this. Yet clinicians and patients are now faced with a rapidly increasing volume of information used for classifying people according to their risk of future outcomes. This has resulted in a shift from diagnosis and treatment of patients based on cut-offs in continuously distributed characteristics (hypertension and cholesterol e.g.) to targeting a patient’s overall risk for future outcomes such as cardiovascular events.[20] GPs in the UK are now routinely using risk calculators for certain conditions such as cardiovascular disease.[21]

Genomics, biological markers and past clinical history – all of these are becoming available in health care datasets in ways which can be used as the basis for this calculation of an individual’s risk of future outcomes. Research investigates whether intervention to change those risks has good or bad effects. This is all about prognosis and draws on the science of cohort studies and risk prediction. It is important that undergraduate and postgraduate students have a grasp of the ideas and interpretation around figures of absolute risk and probability, and the sort of differences which investigations and treatments may make to estimates of benefit and harm and how to discuss these with patients and explore patients’ own valuations of desirable outcomes. Teaching and training already include ‘numbers needed to treat’ and ‘smiley face charts’ as the basis for discussion and communication with patients. This is prognosis-in-practice and offers the opportunity to compare and discuss different outcomes in relation to decisions the patient might make.

The question that remains for medical teaching and training is just how much, when talking and communicating about the future, do any of us – scientists, doctors, patients and public – really understand the notion of probability as applied to the individual. The estimates of likely outcomes are derived from studying groups of patients, and the clinician has to help interpret how these apply to the individual patients in front of him or her. Diagnosis, however unwelcome the news it brings, can provide a welcome degree of certainty for both doctor and patients. It guides practical action, from validating work absence to a surgical operation. Decision-making on the basis of probabilities is more challenging and yet this is what is at the heart of traditional primary care. There is evidence that patients and their doctors are good at predicting outcome in common clinical presentations[22]; statistically derived estimates, from standard protocols or decision aids, add information to this prediction but often not substantially. Yet the language in which probability and risk are discussed, and the task of assimilating and presenting prognostic information in ways that can be used by individual patients and their doctors, remain major challenges for clinical practice and training.

Medical education needs to focus on the ways in which prognostic information about likely outcomes – including diagnosis – can be used and interpreted to support decisions in any consultation, including encounters for which algorithms and guidelines are available, and to rehearse and discuss the process of risk communication with the patient.

## Conclusion

The potential that prognosis might replace diagnosis as the core model of practice does not assume that diagnosis is less important than it was – on the contrary, good prognostic information enhances the usefulness of diagnosis. However, it does mean that medicine should become more demanding and critical of new diagnostic technology and treatments, and students should be engaged in these discussions and debates during their training as they also develop and maintain their confidence in how to diagnose. More importantly, it means that the prognosis question should be asked about patients in primary care as the driver and support for decisions – ‘Given all the information that I have to hand, this is what is likely to happen if we do this rather than that’. Stratified care guidance may indicate what should preferentially be done at the policy level, but information about future probabilities must in the end be about shared decisions with the patient. The education of us all in how to use prognostic data to inform decisions is a major task ahead.

## References

[CIT0001] Del Mar C, Doust J, Glasziou P (2006). Clinical thinking.

[CIT0002] Howie JGR (1972). Diagnosis – the Achilles heel?. J. R. Coll. Gen. Pract..

[CIT0003] Martin SA, Boucher M, Wright JM (2014). Mild hypertension in people at low risk. BMJ.

[CIT0004] Jarvinen TL, Michaelsson K, Jokihaara J (2015). Overdiagnosis of bone fragility in the quest to prevent bone fracture in the quest to prevent hip fracture. BMJ.

[CIT0005] Moynihan R, Doust J, Henry D (2012). Preventing overdiagnosis: how to stop harming the healthy. BMJ.

[CIT0006] Croft P, Altman DG, Deeks JJ (2015). The science of clinical practice: disease diagnosis or patient prognosis? Evidence about “what is likely to happen” should shape clinical practice. BMC Med.

[CIT0007] Dinant G-J, Buntinx FF, Butler CCC (2007). The necessary shift from diagnostic to prognostic research. BMC Fam. Pract..

[CIT0008] Hemingway H, Croft P, Perel P (2013). Prognosis research strategy (PROGRESS) 1: a framework for researching clinical outcomes. BMJ.

[CIT0009] Gawande A (2015). Being mortal.

[CIT0010] Wiener RS, Schwartz LM, Woloshin S (2013). When a test is too good: how CT pulmonary angiograms find pulmonary emboli that do not need to be found. BMJ.

[CIT0011] National Institute for Health and Care Excellence (2014). Atrial fibrillation: the management of atrial fibrillation. NICE clinical guideline 180.

[CIT0012] Downie A, Williams CM, Henschke N (2013). Red flags to screen for malignancy and fracture in patients with low back pain: systematic review. BMJ.

[CIT0013] Hill JC, Whitehurst DG, Lewis M (2011). Comparison of stratified primary care management for low back pain with current best practice (STarT Back): a randomised controlled trial. The Lancet.

[CIT0014] Jarvik JG, Hollingworth W, Martin B (2003). Rapid magnetic resonance imaging vs radiographs for patients with low back pain. JAMA.

[CIT0015] White PD, Goldsmith KA, Johnson AL (2011). Comparison of adaptive pacing therapy, cognitive behaviour therapy, graded exercise therapy, and specialist medical care for chronic fatigue syndrome (PACE): a randomised trial. Lancet.

[CIT0016] Lynch CP, Gebregziabher M, Zhao Y (2014). Impact of medical and psychiatric multi-morbidity on mortality in diabetes: emerging evidence. BMC Endocr. Disord..

[CIT0017] Lewis GN, Rice DA, McNair PJ (2015). Predictors of persistent pain after total knee arthroplasty: a systematic review and meta-analysis. Br. J. Anaesth.

[CIT0018] Barraclough K, Gale CP, Hall R (2015). Assessment of chest pain in a low risk patient: is the exercise tolerance test obsolete?. BMJ.

[CIT0019] Kent DM, Hayward RA (2007). Limitations of applying summary results of clinical trials to individual patients. JAMA.

[CIT0020] Krumholz HM (2013). Target cardiovascular risk rather than cholesterol concentration. BMJ.

[CIT0021] http://www.qrisk.org/.

[CIT0022] Stolper E, Van de Wiel M, Van Royen P (2011). Gut feelings as a third track in general practitioners’ diagnostic reasoning. J. Gen. Intern. Med.

